# Draft genome of *Bacillus velezensis* CMRP6330, a suitable biocontrol agent for disease management in crops

**DOI:** 10.1128/mra.00657-24

**Published:** 2024-11-06

**Authors:** Gustavo Manoel Teixeira, Giovana Camila Cordeiro Montanari, Maria Luiza Abreu Nicoletto, Daniel Vieira da Silva, Sandriele Aparecida Noriler, João Paulo de Oliveira, Marcus Vinicius da Silva Rodrigues, Danilo Sipoli Sanches, Ulisses de Padua Pereira, Ulisses Nunes da Rocha, Admilton Gonçalves de Oliveira

**Affiliations:** 1 Department of Microbiology, Universidade Estadual de Londrina, Londrina, Brazil; 2 Computer Science Department, Universidade Tecnológica Federal do Paraná, Cornélio Procópio, Brazil; 3 Department of Preventive Veterinary Medicine, Universidade Estadual de Londrina, Londrina, Brazil; 4 Department of Applied Microbial Ecology, Helmholtz Centre for Environmental Research–UFZ GmbH, Leipzig, Germany; Rochester Institute of Technology, Rochester, New York, USA

**Keywords:** *Bacillus velezensis*, biocontrol agent, genomics, crop production

## Abstract

As a biological alternative to managing diseases in crop production, we highlight the *Bacillus velezensis* strain LABIM41 (CMRP6330). Its genome, composed of 3,970,959 bp, possesses a rich metabolic machinery and a wide range of molecules with different biological activities and roles in its symbiotic relationship with its plant hosts.

## ANNOUNCEMENT

The search for microorganisms harboring potential bioactive compound machinery has significantly advanced since the popularization of sequencing techniques (1–3). Investigating the genomic content of microorganisms from the most diverse environments has shown an uncovered potential in environments like soil from nutrient-rich places (4, 5), microorganisms from marine environments (6, 7), and endophytic bacteria (8, 9). Soil from well-conserved vegetation in tropical environments is a diverse source of microorganisms (10), which can also be viewed as a source of undiscovered molecules with different biotechnological applications for many areas. Robust and well-curated biological databases allow researchers to investigate genomic data for known and unknown metabolic machinery (11, 12). Using antiSMASH (13), the genomic content of a bacterial isolate from an Atlantic Forest soil sample was explored following its assembly and analyzed in the search for known secondary metabolite machinery.

**Fig 1:**
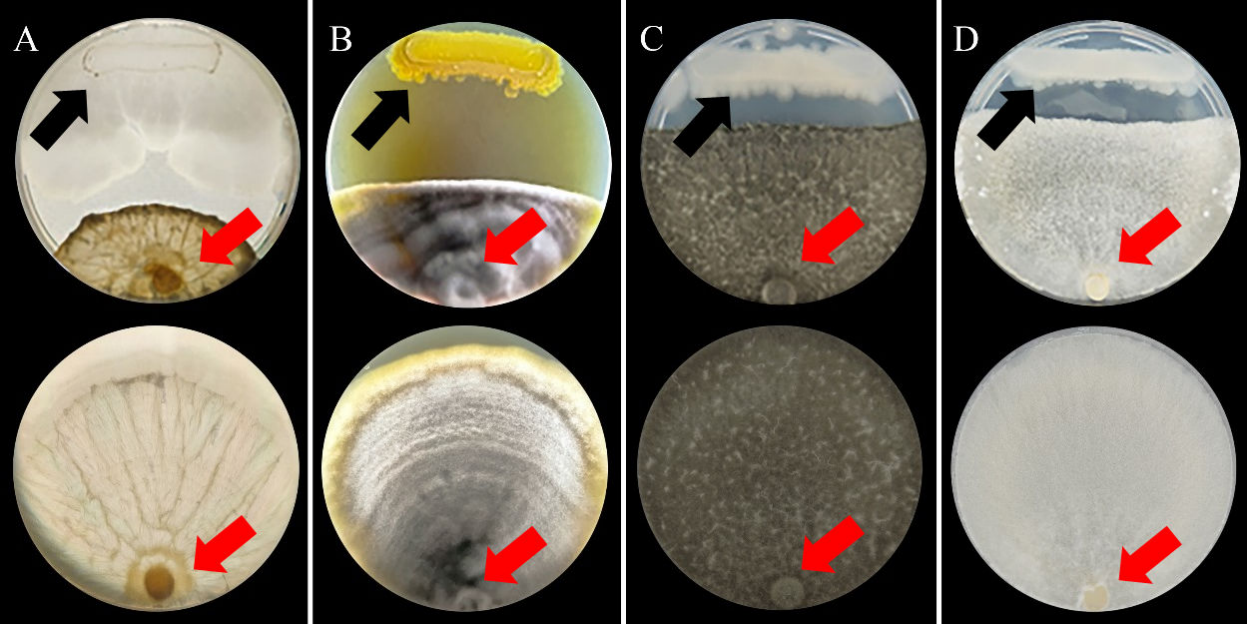
Antagonistic activity of *Bacillus velezensis* CMRP6330 on phytopathogenic fungi mycelial growth. The experiment was conducted on 90-mm plates with PDA culture medium. The fungi used were (**A**) *Colletotrichum truncatum,* (**B**) *Cercospora kikuchii,* (**C**) *Macrophomina phaseolina*, and (**D**) *Sclerotinia sclerotiorum*; the images above show the control of mycelial growth of the respective fungi. For each of the experiments, the top plates show the *B. velezensis* CMRP6330 inoculated (black arrow) with the different fungi (red arrow), and the bottom plates show the growth of the fungi in the absence of *B. velezensis* CMRP6330.

The isolated strain was collected from a soil sample used in a practical activity with students from the Microbiology courses at the State University of Londrina (UEL). Soil samples were collected from a forest fragment (S23,32/W51,20), dissolved in saline solution, shaken for 5 minutes, and heated for 10 minutes at 80°C. Serial dilutions were realized, and aliquots were spread over agar plates, then incubated for 48 hrs at 28°C. Isolated colonies showing clear antagonistic effect to four different pathogenic fungi (*Colletotrichum truncatum*, *Cercospora kikuchii*, *Macrophomina phaseolina*, and *Sclerotinia sclerotiorum*) on Potato Dextrose Agar (PDA) plates were selected for further *in vitro* experiments. The strain with the strongest biological activity was selected (Fig. 1) and deposited in the microorganism’s Laboratory of Microbial Biotechnology (LABIM) collection at UEL as LABIM41 (CMRP6330).

Aiming its sequencing, we cultivated the strain from its stock tube at LABIM for 48 hrs at 28°C in Luria Bertani. For DNA extraction, the Gentra Puregene Genomic DNA (Qiagen Brasil) kit was used following the manufacturer’s instructions. The sequencing reaction was conducted at the Soil Biotechnology Laboratory at the Empresa Brasileira de Pesquisa Agropecuária (EMBRAPA) with an Illumina MiSeq Paired-End 300 bp.

FastQ reads from the sequencing were verified using FastQC v0.11.9 (14) to assess their quality. Using the report from the previous software, trimming parameters were defined and used for raw data filtering with Trimmomatic v0.39 (15). Filtered reads were used for contig assembly using SPAdes v3.13 (16). Different assemblies were compared using QUAST v5.0.2 (17). The best assembly was chosen based on the higher N50 (873,453 bp) and contig size (1,108,929 bp), alongside the fewest contigs (26) and a total assembly size close to the reference genomes (3,950,216 bp). Scaffolds were generated using contigs larger than 500 bp with Medusa (18). The remaining gaps were manually curated using recursive mapping of the reads against the main scaffold and gap shortening with the edges of the reads around the gaps (19, 20). The completed chromosome was annotated using PGAP (21). The assembled genome contained 3,970,959 bp and 3,848 CDS with a G+C content of 46.47%. Species identification using orthoANI (22) revealed that the isolated strain was *Bacillus velezensis*.

The results obtained with antiSMASH confirmed the presence of the genetic machinery capable of producing important molecules with biotechnological applications against phytopathogenic agents, such as fengycins (23–25), surfactins (24, 26), macrolactins (27), and difficidin (28) (Table 1).

**TABLE 1:** List of 13 biosynthesis gene clusters (BGCs) found in the CMRP6330 genome using antiSMASH, alongside its biological properties when available

BGCs—type	Compound type	From	To	Activity
Unidentified 1—thiopeptide, LAP		266,387	296,123	
Surfactin—NRPS	NRP: lipopeptide	306,839	372,367	Biosurfactant with great emulsifying capabilities. Causes damage to membranes (26).
Butirosin A/butirosin B—PKS-like	Saccharide	899,877	941,121	Amynoglycosidic antibiotic with activity against Gram-negative bacteria (29).
Unidentified 4—terpene		1,023,922	1,044,662	
Macrolactin H—transAT-PKS	Polyketide	1,374,077	1,462,313	Cytotoxic and antiviral activities. Highly diverse structurally (27).
Bacillaene—transAT-PKS, T3PKS, NRPS	Polyketide+NRP	1,686,127	1,796,241	Antibiotical activity described. Poorly characterized structurally (30).
Fengycin—NRPS, transAT-PKS, betalactone	NRP	1,855,051	1,992,155	High antifungal activity and capable of inducing plant defense systems (25).
Unidentified 8—terpene		2,015,171	2,037,054	
Unidentified 9—T3PKS		2,120,696	2,161,796	
Difficidin—transAT-PKS	Polyketide	2,281,309	2,387,488	Activity against fire blight (28).
Bacillibactin—NRP-metallophore, NRPS, RiPP-like	NRP	3,020,902	3,072,690	Iron chelator (31).
Bacilysin—other	Other	3,601,278	3,642,696	Activity against fire blight (28).

## Data Availability

This Whole Genome project has been deposited in GenBank under accession no. CP102511.1. Raw reads were deposited at the SRA database under the following accession: SRX21620088. The Bioproject and Biosample accessions are available through the following codes, respectively: PRJNA866538 and SAMN30168657.
